# Judgement and Decision Making in Clinical and Return-to-Sports Decision Making: A Narrative Review

**DOI:** 10.1007/s40279-024-02054-9

**Published:** 2024-06-26

**Authors:** Kate K. Yung, Clare L. Ardern, Fabio R. Serpiello, Sam Robertson

**Affiliations:** 1 Institute for Health and Sport, Victoria University, Melbourne, Australia; 2 Department of Orthopaedics and Traumatology, Faculty of Medicine, The Chinese University of Hong Kong, Shatin, Hong Kong, China; 3 Sport and Exercise Medicine Research Centre, La Trobe University, Melbourne, Australia; 4 Department of Physical Therapy, University of British Columbia, Vancouver, Canada; 5 Human Exercise and Training Lab, School of Health Medical and Applied Sciences, Central Queensland University, Rockhampton, Australia

## Abstract

Making return-to-sport decisions can be complex and multi-faceted, as it requires an evaluation of an individual’s physical, psychological, and social well-being. Specifically, the timing of progression, regression, or return to sport can be difficult to determine due to the multitude of information that needs to be considered by clinicians. With the advent of new sports technology, the increasing volume of data poses a challenge to clinicians in effectively processing and utilising it to enhance the quality of their decisions. To gain a deeper understanding of the mechanisms underlying human decision making and associated biases, this narrative review provides a brief overview of different decision-making models that are relevant to sports rehabilitation settings. Accordingly, decisions can be made intuitively, analytically, and/or with heuristics. This narrative review demonstrates how the decision-making models can be applied in the context of return-to-sport decisions and shed light on strategies that may help clinicians improve decision quality.

## Key Points

**Table:** 

This narrative review offers a brief introduction to decision-making models relevant to sports rehabilitation settings, demonstrating their applications in return-to-sport decisions and providing strategies to improve decision quality.
We discuss the interplay between intuitive and analytical processes in decision making, as well as the influence of adaptations and biases on clinical judgement.
Clinicians are encouraged to adopt the decision-making models that suit the context and environment to enhance their return-to-sport and overall clinical decision-making qualities.

## Introduction

Injuries are an unfortunate reality in sports, and it is crucial to carefully determine when an athlete can safely return to sport (RTS). While existing literature often emphasises the RTS criteria, it is also essential to recognise the significant role of judgement and decision making (JDM) in this complex process. Judgement is a multifaceted process that involves evaluating information, assessing alternatives, and forming opinions or conclusions to inform a decision [1]. It involves the application of personal beliefs, values, and knowledge to interpret and comprehend the available information. Decision making, on the other hand, pertains to the cognitive process of selecting a course of action or making a choice among various alternatives, with the consequences of that choice holding significance [2]. A decision on RTS is when a clinician has to purposely select the best option among a set of alternatives in light of a set of given criteria to decide when the athlete can RTS without any medical restrictions [3]. While good decisions may not necessarily lead to a good decision outcome, RTS judgement and decisions can be challenging as the outcome pertains to the athlete’s well-being and performance [4]. For example, if RTS is delayed for a lesser chance of re-injury, reduced player availability may negatively impact team performance [5, 6]. On the contrary, premature RTS has been suggested as a possible risk factor for re-injury in football codes [7, 8]. The study of JDM has more than 50 years of history and has been influenced by different disciplines such as psychology, economy, and neuroscience, yet its presence in sports rehabilitation has been limited [4, 9, 10].

Competencies required by clinicians for JDM include, but are not limited to, identifying the key factors in a complex situation, and considering the risk(s) and benefit(s) associated with decisions. Research has indicated that clinicians take into account a range of biopsychosocial and contextual factors, including biological healing, playing position, and social support, when making RTS decisions [3, 11]. Despite significant research focused on developing RTS criteria, JDM training is often not included in clinicians' education, and limited attention has been given to JDM in sports rehabilitation. However, JDM is important in daily operations, where scientific evidence and experience-based judgement are both involved in the decision making. Although scientific research values universal validity and methodological rigour, it is unavoidable to have uncertainty in sports rehabilitation due to factors such as sample sizes and multifactorial outcomes. As researchers strive to investigate RTS protocol and exit criteria, it is crucial to grasp the fundamental principles of decision making, such as the use of simple rules and heuristics [9]. As with different approaches, there are pros and cons of any method. For example, the use of rules may lead to faster and more accurate judgement, while at times it may lack science-grade evaluation.

Considering that clinicians play a vital role not only in making judgements and decisions related to RTS, but also in their daily operations and in understanding the behaviours of athletes, coaches, and managers, it is valuable to investigate the significance of JDM in both the RTS process and the broader context of sports clinical settings. With reference to work in other areas, such as in physical education [12], this review provides a brief overview of different decision-making models relevant to sports rehabilitation settings.

### Brief Introduction to Decision Making

Decisions in sports often involve uncertainty due to the dynamics of sport and are typically made under three conditions [13]:

1. Certainty. Decision makers have exact and full information regarding the expected results for the different alternatives at hand.
2. Risk. Decision makers lack complete certainty regarding the outcome of the decision but are aware of the probabilities associated with their occurrence.
3. Uncertainty. Decision makers lack decision alternatives, and their potential outcomes are relatively unknown.

Traditional rational models of JDM assume that decision makers are rational and well informed, and constantly choosing the option with the best outcome [14]. However, this perspective solely focuses on the outcome and represents an ideal condition, which may not always reflect reality. In contrast, Herbert Simon (1957) introduced the concept of bounded rationality, which challenges the traditional assumption that individuals consistently maximise utility and make decisions through deliberate calculations of weighted sums [15]. Due to various constraints, such as cognitive limitations (e.g., lack of knowledge) and environmental factors (e.g., lack of time or resources), Simon suggested that humans often make decisions in a more limited and practical manner [16]. When confronted with complex scenarios in reality, decision makers strive to make rational decisions that are often constrained by different factors. As a result, they often choose the first alternative that satisfies their aspiration levels rather than aiming for maximised or optimised outcomes, which is known as *satisficing.* While classical rationality is considered a normative model, bounded rationality is a type of descriptive model that describes how people actually make decisions [17].

The departure from the notion of full rationality has led to the development of alternative approaches to decision making. Recognising that humans often deviate from rationality in their decision-making processes, researchers have delved into descriptive models that aim to understand and explain these deviations. These models encompass a wide range of aspects, including the study of heuristics and biases. Heuristics are defined as “principles which reduce the complex task of assessing probabilities and predicting values to simpler judgemental operations” (Tversky and Kahneman, 1974, p. 1124) [18]. That is, they are simple decision-making strategies or mental shortcuts that individuals use to make judgements quickly and efficiently. In their intriguing work on heuristics and biases, Daniel Kahneman and his colleagues proposed that individuals often rely on feelings of representativeness and availability when making automatic probability judgements [18]. Additionally, they may inadvertently anchor their judgements on potentially irrelevant information [18]. Sometimes, these shortcuts may lead to systematic deviations in decision making (i.e., bias) [19], as they may oversimplify complex problems or important information.

However, within the realm of naturalistic decision-making approaches, a seemingly contradictory view on heuristics has emerged [20]. Key among the proponents of this view is psychologist Gerd Gigerenzer, who has advanced the concept of the simple heuristics approach with the ‘adaptive toolbox’ [21]. The adaptive toolbox is a concept within the framework of the simple heuristics approach. It suggests that decision makers possess a repertoire of simple decision rules or heuristics that they can draw upon to make judgements and choices in different situations. These heuristics are considered adaptive and show that people adapt to their constraints and rely on the structure of the environment [18]. The adaptive toolbox view contrasts with the heuristics and biases framework put forth by Tversky and Kahneman [18]. While both perspectives acknowledge the role of heuristics in decision making, they differ in their interpretations. The heuristics and biases framework, developed by Tversky and Kahneman [18], focuses on identifying cognitive biases and limitations in decision making. It highlights instances where heuristics can lead to systematic errors and deviations from rationality (i.e., bias). This framework emphasises the potential pitfalls of relying on heuristics and aims to uncover the biases they may introduce. In contrast, Gigerenzer's adaptive toolbox [21], or simple heuristics, takes a more positive view of heuristics. It suggests that heuristics are not necessarily flawed or biased but rather adaptive responses to the constraints of decision-making environments [21]. It is an adaptive mental toolbox that is packed with simple but accurate tools for making decisions under uncertainty. The adaptive toolbox framework emphasises the efficiency, effectiveness, and adaptability of heuristics in achieving satisfactory decision outcomes.

Building on the insights of bounded rationality and heuristics and biases, researchers proposed a seemingly relevant yet distinct model for decision making, which is known as the dual-process model (DPM). DPM systematically encompasses both intuitive and analytical processes in decision making, which are referred to as System 1 and System 2, respectively [22, 23]. System 1 involves intuitive decisions, while System 2 involves systematic, analytical decision making. The DPM has emerged as a descriptive framework that incorporates insights from cognitive psychology, behavioral economics, and neuroscience. It acknowledges the limitations of rational decision making and recognises the role of automatic, intuitive processes alongside deliberate, analytical thinking. The theory and practice of DPM have been used extensively in different contexts, including medical science [24, 25], but have yet to be widely adopted in the realm of sports medicine.

In this narrative review, we aim to advance our understanding of this important field by exploring the two distinct decision-making models: heuristics and biases and the DPM. In the following sections, we first discuss the heuristic and bias approach by Tversky and Kahneman [18], followed by the adaptative toolbox proposed by Gigerenzer [21]. Then, we discuss the DPM, the interplay between the two systems, and more importantly, their relevance to sports clinical settings. Finally, we explore how clinicians can minimise the impact of potential biases and adopt mitigating strategies. A glossary of terms relevant to JDM can be found in Table 1.

**Table 1:** A glossary of terms in judgement and decision making

Term	Definition and concept
Bounded rationality	When decision making is bounded by the cognitive constraints (e.g., knowledge limitation) and environmental constraints (e.g., time or resources available to decision makers) [26]
Judgement	The process of evaluating information, assessing alternatives, and forming opinions or conclusions to guide a decision. It involves applying personal beliefs, values, and knowledge to interpret and make sense of the available information [27]
Decision making	The process of selecting a choice from a range of options, with the consequence of that choice being important [2]
Choice	Outcome of the judgement and decision making [1]
Certainty	When the decision maker has exact and full information regarding the expect results for the different alternatives at hand [13]
Risk	When the decision maker lacks complete certainty regarding the outcome of the decision but is aware of the probabilities associated with their occurrence [13]
Uncertainty	When the decision alternatives and their potential outcomes are relatively unknown [13]
Heuristics	Mental shortcuts or rules of thumb that individuals use to simplify the decision-making process [18]. The decision is based on a subset of all available information
Bias	Systematic deviations from rationality in judgement and decision making [18]
Intuition	A form of decision making or judgement that operates automatically and effortlessly, often without conscious awareness or deliberation [28]. The decision may be based on all of the available cues

## Heuristics and Bias

Kahneman and Tversky’s research on heuristics and biases has shed light on the underlying cognitive processes and their impact on decision making [18, 29]. Heuristics are mental shortcuts or rules of thumb that individuals use to simplify the decision-making process [18]. That is, judgement under uncertainty may often rely on a few simplifying heuristics rather than extensive algorithmic processing [29]. At times, the use of heuristics may compromise the outcome of decision making, leading to systematic deviations from rationality (i.e., bias) [30]. There are three main types of heuristics and bias, which are anchoring, availability, and representativeness [18]. In the context of RTS, we highlight some common cognitive biases that may occur.

### Anchoring

*Description:* Anchoring is when decision makers are ‘anchored’ on the initial values and later update their perception with better information [31]. Accordingly, decision makers tend to fixate on the first impression of a clinical case, such as some specific clinical features early on in the diagnostic process (anchor) [32]. Following the initial piece of information, interpretations are made around the anchor. This can be an effective strategy in a busy clinic as it allows clinicians to give fast and likely accurate judgement. Yet, at times, clinicians may fail to adjust the hypothesis sufficiently in light of subsequent information.

*Clinical example:* Internal medicine residents use anchoring when they estimate the probability of a disease by using a high or low anchor as the starting point [33].

*Anchoring bias* occurs when the decision maker relies heavily on the initial information (anchor) offered to make a judgement [34].

*Clinical example:* A lacrosse player is hit on the ribs with a lacrosse stick, with signs of bruising. The player was able to continue playing afterwards. A clinician may anchor on the initial piece of information (a contact bruise injury) and neglect the subsequent information that there was significant localised swelling. In this case, the clinician may have missed a rib fracture injury and wrongly estimated the time to RTS.

### Availability

*Description:* Availability heuristics is the mental shortcut that relies on the most readily available data that comes to the person’s mind when evaluating a decision, topic, or event. This is because people have a tendency to place greater weight on information that can be easily remembered and quickly retrieved [35].

*Clinical example:* An athlete with a syndesmosis injury may estimate their recovery time based on a teammate’s recent experience with the same injury. However, the accuracy of this heuristic can be influenced by the recentness and vividness of memories [31]. It may lead to availability bias if the decision maker disregards information that does not support the belief.

*Availability bias* is the cognitive bias associated with availability heuristics, in which a decision maker tends to rely on immediate examples that readily come to mind [35]. Accordingly, decision makers would perceive the most readily available evidence as the most relevant and important [35].

*Clinical example:* When a clinician has just finished seeing an athlete with muscle soreness due to recent high-intensity training in the sports club, the clinician may perceive the next athlete coming in with muscle soreness as having the same issue. However, that athlete may, in fact, be suffering from a low-grade muscle strain injury from a different injury mechanism. Inexperienced clinicians, driven by the availability bias, tend to rely on readily available common prototypes. Conversely, experienced clinicians are more inclined to consider atypical cases, broadening their diagnostic considerations and reducing the impact of the availability bias [36]. Clinicians can enhance their judgement by engaging in reflective reasoning [37, 38].

### Representativeness

*Description:* Representative heuristics is usually used when individuals are asked to assess the likelihood of an object or event belonging to a specific class or process. Individuals often categorise by matching the similarity of an object or incident to an existing one that has already existed in their minds [18].

*Clinical example:* When a clinician encounters a patient with classic symptoms of a well-known and frequently occurring condition, the availability heuristic allows for quick recognition and diagnosis. For example, recognising the immediate signs and symptoms of a heart attack (e.g., chest pain, shortness of breath) can prompt clinicians to initiate appropriate interventions promptly [39].

*Representative bias* is when this heuristic can lead to disproportionate evaluations of events, primarily influenced by emotional biases. These biases can significantly impact the accuracy of event assessments, ultimately resulting in erroneous decisions.

*Clinical example:* Clinicians may be biased towards diagnosing and treating injuries that are prevalent in a particular sport or position. For example, in football, where ankle sprains are common, a clinician may attribute ankle pain to a lateral ankle sprain rather than considering alternative diagnoses.

## Simple Heuristics

Over the last 40 years, cognitive psychologists have identified more than 100 known biases [32, 40]. Along with some other researchers, psychologist Gigerenzer argues that heuristics should be viewed as the human mind’s ‘adaptive toolbox’ that allows a person to associate new information with existing patterns or thoughts [41–43]. Based on the view of the adaptative toolbox, heuristics are a shortcut to an automatic brain. While employing them may demand conscious effort, it is crucial to recognise that heuristics should not be automatically deemed inferior to other decision-making strategies solely because they are mental shortcuts [44–47]. Accordingly, there are three major building blocks for a heuristic: the search rule, the stopping rule, and the decision rule [45]. The search rule refers to the strategy or process used to gather information or explore the available options. The stopping rule determines when the search for information or exploration of options should cease. The decision rule is the guideline or criterion used to evaluate the gathered information and determine the final choice. Together, these three components—search rule, stopping rule, and decision rule—form a framework that helps individuals navigate the decision-making process by providing guidance on information acquisition, determining when to stop gathering information, and ultimately making a choice based on the evaluated information (see Gigerenzer and Gaissmaier for an in-depth review of the topic [41]). In certain circumstances, a simple decision strategy with less information input may outperform deliberate reasoning via detailed analyses [48–51].

The use of heuristics has been studied in diverse domains, such as psychology [45], law [52], sports [53, 54], medicine [55, 56], finance [57], and political science [58]. In medicine, using heuristics can help clinicians make accurate, transparent, and quick decisions [32, 55], yet only limited research is available in the field of RTS [59]. Heuristics can also be utilised to improve sports safety. For example, clinicians can educate a parent-coach on how to assess injuries at the pitch side by following a structured approach: conducting a set of tests in a specific order (search rule), recognising which parameter indicates a critical condition that necessitates immediate action (stopping rule), and knowing the appropriate course of action when such a condition is identified (e.g., referring the individual to the emergency department). As heuristics are adaptive in nature, they are neither good nor bad per se if applied appropriately in situations where they have been adopted. The following are several examples of heuristics in the context of RTS.

### Take-the-Best

*Description:* Take-the-best refers to a situation where decision makers search through the alternatives in order of validity and base the choice on the ‘best’ option [41].

*Clinical example:* A clinician may evaluate an athlete’s fitness for RTS by considering the best available indicators such as running speed, strength, and mental preparedness.

### Elimination by Aspects

*Description:* Elimination by aspects is when a decision maker reduces the alternatives by eliminating those that do not meet the required criteria for a specific attribute [60].

*Clinical example*: When a clinician prescribes exercise for an athlete with a tibia stress fracture, the clinician will first compare a selection of exercises on the lower limb and eliminate the weight-bearing ones.

### Fast-and-Frugal Trees

*Description:* A fast-and-frugal tree is similar to a decision tree, where decision makers classify and decide quickly with a few attributes [41]. There has been a range of applications in different fields, for example, clinicians determining if a patient with severe chest pain has a heart attack or not [61], and magistrates making bail decisions in court [52]. In orthopaedics, clinicians can use the Ottawa Ankle Rules to decide whether an injured ankle requires an X-ray to rule out a fracture [62]. Ottawa Ankle Rules have successfully been implemented in applied settings, reducing unnecessary radiographs by 30–40% [63].

**Fig. 1:**
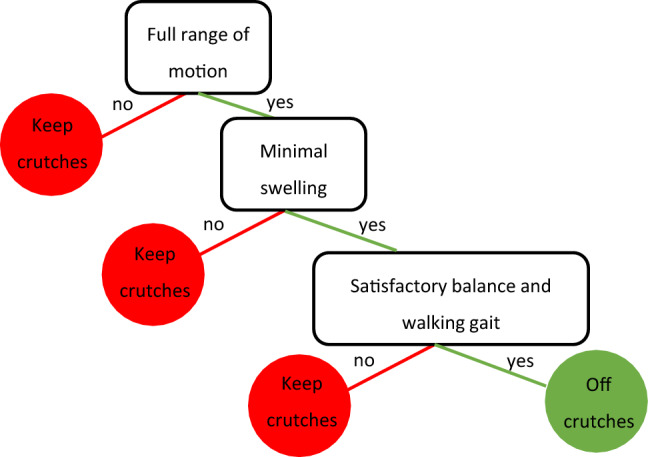
Fast-and-frugal tree to decide if an athlete can walk without crutches

*Clinical example:* Clinicians may use a fast-and-frugal tree to decide whether an athlete may walk without crutches after an anterior cruciate ligament reconstruction surgery (Fig. 1).

### Confirmation Heuristics

*Description:* Humans tend to search for, interpret, favour, and recall information that validates their pre-existing beliefs or hypotheses [64]. That is, we naturally look for evidence that is supportive of the hypotheses we favor and seldom seek evidence naturally to falsify the hypothesis [65].

*Clinical example:* An athlete visits a clinician to consult for her prolonged anterior shin pain. Based on clinical reasoning, the clinician promptly forms an initial hypothesis regarding the underlying cause (tibial stress fracture). This hypothesis guides the clinician’s search for relevant information, such as ordering clinical tests and assessing risk factors for relative energy deficiency in sports, with a focus on confirming the hypothesis.

### Sutton’s Law

*Description:* Sutton’s Law in clinical reasoning refers to prioritising tests that have a higher diagnostic value and as such, focusing efforts on the most apparent diagnosis when allocating resources [66]. It is effective and preferred in many cases. Occasionally, Sutton’s Slip may occur, which is a missed or delayed diagnosis of less common but significant conditions, such as oncologic conditions [67].

*Clinical example:* An athlete consults a clinician for prolonged low back pain. One possible and apparent diagnosis is lumbar spine sprain or strain, with other differential diagnoses including discogenic pain, spondylolysis, and oncologic condition. The clinician focused on the most possible diagnosis and ordered radiographs (X-ray and magnetic resonance imaging) for the low back. It was later found to be metastatic breast cancer [68].

### Framing Effect

*Description:* Humans may be susceptible to how others frame the options, known as the ‘framing effect’ [69]. Different phrasing ways can change a neutral message to an implicit recommendation and affect one’s decision, such as treatment selections [70]. For example, patients are more inclined to consider surgery when the clinician uses a survival frame rather than a mortality one, although they are logically equivalent [71]. The framing effect may vary with the type of scenario and the responder’s characteristics.

*Clinical example:* The way a clinician frames the chance of reinjury may affect the athlete’s perception of when to RTS. Fortunately, the framing effect tends to disappear when complete information is provided and expressed in multiple ways [70, 71].

## Dual Process Model

Heuristics have been extensively studied as specific decision-making strategies that simplify complex problems. As clinicians engaged in daily operations involving various complex decisions, it is valuable to broaden our understanding beyond specific strategies and delve into a comprehensive theoretical framework that encompasses the cognitive processes involved in decision making and their interaction. DPM, which systematically incorporates both intuitive (System 1) and analytical (System 2) processes in decision making, has been widely discussed and examined in depth in the classic book *Thinking, Fast and Slow* by Kahneman [28]. This section aims to provide a brief introduction to the DPM, focusing on its relevance to clinicians. Emphasis will be placed on the dynamic interplay between the two systems within the model in the context of RTS and the warning signs that indicate clinicians have to consider switching the systems. By recognising the suitability of each system in a given situation, clinicians can enhance their ability to discern the most effective decision-making strategy. For instance, heuristics might be more suitable for rapid, intuitive decisions, while complex or novel situations may require analytical thinking. Moreover, understanding these concepts facilitates self-reflection on the decision-making processes and enables clinicians to identify instances where they may overly rely on heuristics, when further analysis might be necessary, or when biases may influence our judgements.

In the DPM, System 1 decision making is characterised by an intuitive approach based on a rapid selection of options without systematic evaluation [41, 72]. System 1 refers to a wide diversity of autonomous processes, such as intuitions, heuristics, and pattern recognition. In other words, it is a form of decision making or judgement that operates automatically and effortlessly, often without conscious awareness or deliberation [28]. Intuition is composed of different cognitive mechanisms and can be learnt in multiple ways, including the accumulation of experience [73]. For instance, over the course of many years working on the pitch-side, clinicians can learn the correlations and formed associations between several cues (e.g., visible symptoms—an injured athlete in pain and holding on to her elbow), and the related diagnosis (e.g., a potential shoulder dislocation). Therefore, upon encountering these cues, experienced clinicians may find the decision process may be relatively simple and require less working memory. These cognitive processes enable clinicians to promptly assess and respond to familiar situations without the need for extensive analytical deliberation. Given the time constraints and pressure often encountered in sports medicine settings, the efficiency and speed of System 1 processing are of particular importance. Similar to heuristics and bias discussed earlier, System 1 may also be susceptible to cognitive biases and errors. Among different biases, anchoring bias, availability bias, and confirmation bias are among the cognitive pitfalls that clinicians should be vigilant about when relying predominantly on intuitive judgements [40]. Awareness of these biases can help clinicians navigate potential errors and enhance the quality of decision making.

Compared with System 1, System 2 is a deliberate, conscious and controlled process characterised by rational thinking [74]. System 2, also known as explicit cognition, involves logical judgement and a mental search for additional information [75]. System 2 may be engaged when clinicians need to analyse data to support clinical decisions. For example, when a clinician diagnoses a sports injury with atypical signs and symptoms, System 2 may be required. System 2 is analytical and follows explicit computation rules, such as adhering to the rationality criteria of expected utility theory [76, 77]. The expected utility theory is a decision-making model considering the expected value of different options and the probability of each outcome [78, 79]. It illustrates how one decides in uncertain conditions based on the outcomes of different options and the probability of each outcome [78, 79]. It presumes that a decision maker will make a rational choice based on evaluating the costs and benefits associated with each option [80, 81]. In this theory, a clinician’s decision is determined by the subjective value assigned to each potential outcome and the estimated likelihood of each outcome [78, 79]. Based on DPM, System 2 may have the best outcome if individuals are rational and have access to information about the probabilities and consequences of each option in terms of time, resources, and knowledge [11]. Detailed examples of the application of the expected utility theory in sports injury can be found in the literature [4].

Various characteristics have been attributed to Systems 1 and 2 (see Table 2). However, it is important to note that not all of these characteristics are necessary or defining (for debate on the characteristics of the systems, see Evans and Stanovich [23]). While not conclusive, in general, System 1 is more autonomous (e.g., to work through the decision tree and recognise injury patterns), while System 2 involves cognitive decoupling and hypothetical thinking (e.g., to analyse all available data to decide on RTS medical clearance) [23].

**Table 2:** Comparison of dual process model: System 1 and System 2 approaches in decision making

Typical correlates	System 1 (intuitive)	System 2 (analytic)
Cognitive style	Intuitive Heuristic	Analytical Normative
Operation	Associative	Deductive
Processing	Parallel	Serial
Conscious control/effort	Less	More
Automaticity	Higher	Lower
Reliability	May vary	More consistent
Emotional valence	More	Less
Detail on judgement	Less	More

### Interaction Between the Systems

**Fig. 2:**
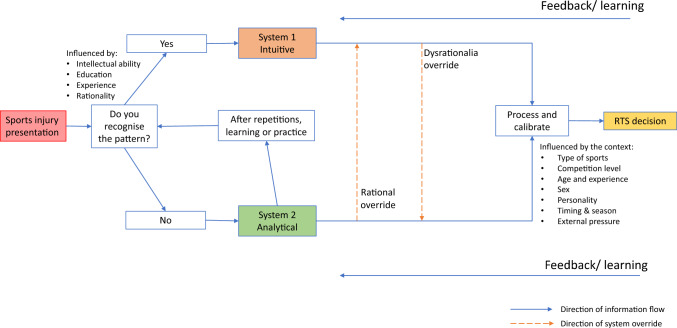
Schematic model for dual process model in the context of return-to-sport decision making

System 2, although known for being more reliable and rational, typically consumes more cognitive resources and longer time. As a result, it may not always be feasible for clinicians to engage in extensive cognitive analysis for every clinical decision they make. Consequently, clinicians may naturally opt for System 1, which is quicker and less demanding on the mind [82]. In some clinical conditions, clinicians may start diagnosing using System 1 based on pattern recognition [83]. For instance, consider a scenario where an athlete presents with acute knee pain and swelling after a sudden twisting motion during a football match. Experienced sports clinicians, attuned to established injury patterns, may swiftly recognise the indicative signs of an anterior cruciate ligament (ACL) injury. These signs include immediate pain, an audible popping sound at the time of injury, joint instability, and the subsequent onset of localised swelling within the knee joint. Additionally, the athlete may report subjective sensations of the knee “giving way” or experiencing instability during physical activity. Leveraging their knowledge and experience, clinicians adeptly connect these symptoms to the distinct pattern associated with an ACL injury. This pattern recognition facilitates the formulation of an initial diagnostic impression, guiding subsequent evaluation strategies such as targeted physical examination manoeuvres, imaging modalities (e.g., magnetic resonance imaging), or appropriate referrals to specialists for definitive confirmation and tailored management. However, when clinicians cannot recognise the pattern (e.g., when athletes cannot recall the exact injury mechanism), they may switch to System 2, which is the deliberate and conscious thought process [84]. In the context of RTS, clinicians may also switch to System 2 in complex conditions, such as when an athlete is eager to participate in an important game despite not being fully healed from an injury (see Fig. 2).

There are also several ways in which the two systems interact, as indicated by the broken orange lines in Fig. 2. The analytical approach of System 2, when used repeatedly, can eventually become automatic, much like the intuitive approach of System 1 [83, 85, 86]. This is analogous to building up sports taping skills, where after considerable practice, the clinician can tape an ankle with little conscious effort. This shows the importance of building up experience and familiarity with clinical practice. With relevant experience, System 1 processing can lead to correct answers in some cases.

System 2 can rationalise and override the intuitive output of System 1 (rational override) [82]. This overriding function requires deliberate mental effort, and the ability to do this can be negatively impacted by distraction, sleep deprivation, and fatigue [87]. Distractions, such as external stimuli or competing thoughts, can divert attention and compromise the ability of System 2 to exert deliberate mental effort. An illustration of this can be observed when a clinician finds themselves making judgements for an athlete on the sidelines, while simultaneously needing to remain aware of the ongoing events happening on the pitch. Similarly, sleep deprivation and fatigue, for example from demanding work schedules and late-night games, can impair cognitive functioning. These factors make it more challenging to engage in reflective thinking and exercise rational override. Consequently, even deliberate thinking can be prone to errors and incorrect answers. Moreover, shallow processing, which involves relying on superficial cues or heuristics, can further impede the ability of System 2 to generate accurate responses. The reliance on shallow processing can lead to erroneous conclusions or judgements, particularly when relevant information or deeper analysis is neglected. It is essential to recognise the limitations posed by fatigue, shallow processing, and insufficient knowledge, as they can all contribute to flawed decision-making processes [22].

System 1 can also override System 2, in which the decision maker overrides a rational judgement based on intuitive feeling, known as dysrationalia [88]. There are several factors that can contribute to dysrationalia. Habitual practices, deeply ingrained beliefs, and personal biases can influence decision-making processes, causing individuals to rely on intuition rather than engaging in deliberate analysis. Emotions in sports, such as fear, excitement, or attachment to specific outcomes, may also play a role in overriding rational judgements. Additionally, the context in which decisions are made can impact the extent to which System 1 overrides System 2. Time pressure, social influence, or the desire to conform to norms are examples of contextual factors that can lead to dysrationalia. An example of System 1 overriding System 2 can be observed in the field of healthcare. Despite the availability of well-developed clinical decision guidelines, clinicians may sometimes deviate from them and persist with certain clinical practices that lack solid evidence [85]. This deviation can stem from various factors, including professional experience, personal beliefs, or the influence of patient preferences. In these instances, intuitive feelings and contextual factors may override the rational judgement based on the guidelines, leading to dysrationalia.

There is a debate in the literature about whether Systems 1 and 2 are qualitatively distinct or should be considered as a continuum. For example, Evans and Stanovich suggested that individuals may use a mixture of two kinds of processing to control how they respond [89], and the degree of mixture differs across individuals. That is, an individual can rely on System 1 for processing, and/or invoke System 2 to confirm the intuition or intervene with System 2 processing for a different answer. Alternatively, there is a viewpoint that Systems 1 and 2 should be seen as part of a cognitive continuum. The cognitive continuum theory (CCT) suggests that the use of analytical and intuitive approaches falls along a spectrum rather than being discrete categories [90]. This theory proposes that individuals adapt their cogitation strategy based on task features and progress, employing a range of cognitive processes that lie between purely intuitive and purely analytical approaches. Understanding CCT can shed light on how individuals navigate between intuitive and analytical thinking, and how they adjust their cognitive strategies based on the demands of a particular situation. As a result, it may increase the transparency of the decision-making process [91].

**Fig. 3:**
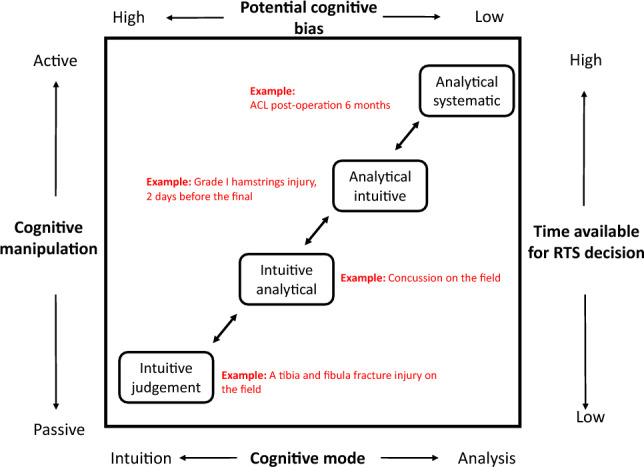
Cognitive continuum theory adapted to the context of return-to-sport (RTS)

Figure 3 illustrates the CCT model of human judgement and decision making adapted to the sports rehabilitation context. The modes of inquiry can be positioned along the continuum based on the degree of cognitive activity they are predicted to induce, such as task structure, cognitive control, and time required [90]. For example, in sports rehabilitation, clinicians may use different modes based on the following scenarios:

- *1. Intuitive judgement: Managing an on-field fracture injury.*
  When an apparent fracture injury (e.g., a tibia and fibula fracture) occurs on-field during a football game, the immediate response of a clinician is to remove the player from the field and send the player to the hospital. This is an intuitive judgement because the clinician is unlikely to allow the injured player to return to the game with a fracture injury due to safety reasons. The time available for decision is short, and the degree of cognitive manipulation is low.
- *2. Intuitive analytical: RTS from a concussion.*
  In case of a suspected concussion during a football game, a clinician will remove the player from the field and assess the player for any subtle change in response, such as facial expression and emotional changes [92]. Clinicians may also use a decision aid (e.g., Sport Concussion Assessment Tool [SCAT6]) to evaluate the concussion at the sideline) [93]. In this case, the time available for the decision is longer than the previous condition (e.g., 5–10 min), and the degree of cognitive manipulation is higher. There is also some degree of intuition (e.g., to observe subtle changes in the player’s response) and analytic involvement (e.g., to assess the condition with SCAT6).
- *3. Analytical intuitive: RTS for a Grade 1 hamstring injury.*
  For a player who sustained a grade 1 hamstring injury two days before the final, a clinician can take the time to assess the player physically, functionally, and mentally. The clinician can decide on RTS based on the assessments. However, due to the limited time frame available for rehabilitation and uncertainties surrounding the player's recovery, a certain degree of intuition may come into play when making the judgments.
- *4. Analytical systematic: RTS for an ACL reconstruction surgery.*
  In the context of ACL rehabilitation, clinicians typically have a longer timeframe, often measured in weeks, to assess and make decisions regarding RTS. During this period, clinicians have the opportunity to conduct comprehensive assessments and perform relevant RTS tests, allowing for a systematic analysis of the results. There is a high degree of cognitive manipulation, and the reliance on intuition may be minimal.

From a practical standpoint, it may not be feasible or even possible to develop a singular model that universally applies to all decision-making scenarios. The complexity and variability of real-world situations often necessitate the consideration and adaptation of multiple decision-making models or approaches. Clinicians, therefore, may draw upon the knowledge of heuristics, DPM, and other models to effectively address the diverse challenges they encounter in their practice.

In short, the knowledge of DPM allows clinicians to scrutinise the underlying decision-making process and realise the systems’ vulnerable aspects. Despite most errors occurring in System 1 [18], it is still valuable to use System 1 in some contexts, for higher efficiency and resources. Both systems are essential for clinicians to function in the applied sports environment. One of the keys to an improved decision-making process is a well-calibrated balance between the two. It is worth noting that decision-making processes are often more complex and can involve interactions between both systems. Therefore, in practice, individuals often rely on a combination of Systems 1 and 2 thinking and depend on the specific context, time pressure, expertise, and personal factors.

## Strategies to Improve Decision Making

Generally, humans are assumed to be rational and to prefer making objective decisions [94]. Clinicians may choose to trust their intuitions when confronted with familiar scenarios that align with their expertise and experiences. Intuition, often associated with System 1 thinking, capitalises on rapid pattern recognition and automatic processing, enabling clinicians to make accurate decisions efficiently. When the clinical presentation matches well-established patterns, heuristics can serve as valuable decision-making shortcuts. For example, when diagnosing common sports injuries, clinicians may rely on recognised symptom clusters and observable patterns to reach accurate conclusions swiftly. However, caution is warranted when applying heuristics and relying solely on intuitive judgements. In complex or novel situations where the patterns may be ambiguous or incomplete, clinicians should pause and consider engaging System 2 thinking. This deliberate and analytical thought process allows for a more comprehensive evaluation of the available information, reducing the influence of biases and increasing the accuracy of decision making.

There is also a tendency among humans to exhibit excessive confidence in decision making, with one of the reasons attributed to blind spot bias [95]. When conducting evaluations of their decision-making process, humans may tend to think they are smarter and less susceptible to cognitive biases than others [96]. In a study by Scopelliti and colleagues, only one out of 661 people said they were more biased than the average [97]. Furthermore, individuals with a pronounced blind spot bias are particularly reluctant to employ strategies aimed at enhancing the quality of their decisions [97]. Given the inherent inclination for humans to be overly confident in decision making, potentially leading to detrimental effects on decision quality, it is beneficial for clinicians to consider using techniques to mitigate the impact where possible, such as incorporating decision aids and increasing self-awareness [40].

Decision aids are useful to improve decision quality. For example, clinicians can use the SCAT6 to aid in assessing, diagnosing, and managing concussions [93]. SCAT6 integrates validated assessment tools, symptom checklists, and step-by-step protocols, providing clinicians with the necessary support to make well-informed decisions regarding diagnosis, treatment, and RTS timelines. Practically, clinicians can conveniently carry a flashcard version of the SCAT6 in their medical bag, ensuring they have quick access to the tool during fast-paced and high-pressure situations on the field. By relying on evidence-based guidelines, clinicians can make confident decisions while effectively managing the complexities of concussion evaluation and care.

In regards to raising awareness, it is a valuable practice for clinicians to constantly reflect on their thought process before deciding and to have the cognitive capacity to decouple from the bias [98]. Specifically, clinicians should be attentive to warning signs that suggest the need to override intuitive responses or heuristics. These signs may include conflicting information, atypical clinical presentations, or situations where the stakes are high, such as RTS decisions involving potential long-term consequences for athletes. Furthermore, clinicians could improve awareness of conditions that may increase their susceptibility to cognitive biases, such as distractions, fatigue, sleep deprivation, and cognitive overload [99]. In such cases, in the context of DPM, clinicians may consider switching from the intuitive processing of System 1 to the analytical processing of System 2, allowing for a more thorough examination and verification of the initial intuition [34].

There are other factors that may also influence the decision quality, such as limited information or emotions [96, 100–102]. These motives and emotions may be intertwined in the decision-making process unintentionally and unconsciously and shape the clinician’s decision [103]. For example, a person feeling anxious about the potential outcome of a risky choice may choose a safer option rather than a risky but potentially lucrative option [104]. The effect of emotional states may also cause decision makers to avoid negative feelings (e.g., guilt and regret) or increase positive feelings (e.g., pride and happiness) [104]. To minimise the magnitude of the emotional effect on the decision process, decision makers can adopt strategies such as time delay, suppression and reappraisal [104]. One of the simplest strategies to minimise the influence of emotions is time delay, which allows time to pass before making a decision. Emotions, including physiological responses, are often short-lived and transient [105]. Most individuals have the adaptability to regulate their emotional states and restore them towards baseline after traumatic events [93].

Suppression is the conscious effort to inhibit emotional responses during the decision-making process, i.e., to suppress or hold back emotional reactions, particularly negative emotions, to maintain clarity and rationality. There are different techniques to help manage and regulate emotional responses, such as deep breathing, mindfulness, or cognitive reframing. The objective is for clinicians to focus more objectively on the facts, data, and logical reasoning involved in the decision.

Reappraisal involves actively reframing or reinterpreting the meaning of an emotionally charged situation. Instead of perceiving a situation solely through an emotional lens, clinicians may try to consciously reinterpret the event in a more objective or positive light. This cognitive reappraisal helps reduce the intensity of negative emotions and allows clinicians to make more balanced and rational judgements. There are a range of strategies that may facilitate better decision making in sports medicine settings and they are summarised in Table 3.

**Table 3:** Strategies and examples for improving decision making

Strategy	Explanation	Examples
Structured data acquisition	Deliberate data acquisition procedure to ensure adequate information is acquired and to minimise blind spots	Use a differential diagnosis checklist tool to assist clinical reasoning [106]
Consider alternatives	Establish routine consideration of alternative options	Seek evidence that may support a RTS decision opposite to the initial impression to force consideration of other examples
Group decision strategy	Seek others’ opinions and apply crowd wisdom	Schedule team meetings with other practitioners and design a rehabilitation plan together
Use of external aid	Improve judgement accuracy by using clinical practice guidelines and algorithms to reduce reliance on memory. Clinicians may also consider the use of clinical decision rules and aids that minimise uncertainty and cognitive load, such as implementing computerised clinical decision support	Visually display a set of clinical tests in the treatment room that clinicians must perform when deciding when an athlete can RTS
Minimise time pressure	Allow adequate time for thought processes	Allow enough time for making a diagnosis and planning for RTS
Supportive environment	Create a supportive environment that encourages high-quality decision making	Ready availability of rehabilitation protocols, clinical guidelines, and RTS criteria to reduce variance Well-organised working schedule to avoid cognitive overload, fatigue and sleep deprivation [99]

## Conclusions

This review serves as an introductory exploration of decision making and its significance for clinicians. It highlights the dynamic interplay between intuitive and analytical processes in decision making, as well as the potential for adaptations and biases to influence clinical judgement. We encourage clinicians to delve into the DPM and other decision-making approaches to enhance their RTS and clinical decision-making abilities. The heuristics and bias and DPM present a valuable model for comprehending clinical reasoning, providing a foundation for future medical education and practice research.
